# Trends and Adaptive Optimal Set Points of CD4^+^ Count Clinical Covariates at Each Phase of the HIV Disease Progression

**DOI:** 10.1155/2020/1379676

**Published:** 2020-03-01

**Authors:** Partson Tinarwo, Temesgen Zewotir, Delia North

**Affiliations:** School of Mathematics, Statistics and Computer Science, University of KwaZulu-Natal, Durban 4000, South Africa

## Abstract

In response to invasion by the human immunodeficiency virus (HIV), the self-regulatory immune system attempts to restore the CD4^+^ count fluctuations. Consequently, many clinical covariates are bound to adapt too, but little is known about their corresponding new optimal set points. It has been reported that there exist few strongest clinical covariates of the CD4^+^ count. The objective of this study is to harness them for a streamlined application of multidimensional viewing lens (statistical models) to zoom into the behavioural patterns of the adaptive optimal set points. We further postulated that the optimal set points of some of the strongest covariates are possibly controlled by dietary conditions or otherwise to enhance the CD4^+^ count. This study investigated post-HIV infection (acute to therapy phases) records of 237 patients involving repeated measurements of 17 CD4^+^ count clinical covariates that were found to be the strongest. The overall trends showed either downwards, upwards, or irregular behaviour. Phase-specific trends were mostly different and unimaginable, with LDH and red blood cells producing the most complex CD4^+^ count behaviour. The approximate optimal set points for dietary-related covariates were total protein 60–100 g/L (acute phase), <85 g/L (early phase), <75 g/L (established phase), and >85 g/L (ART phase), whilst albumin approx. 30–50 g/L (acute), >45 g/L (early and established), and <37 g/L (ART). Sodium was desirable at approx. <45 mEq/L (acute and early), <132 mEq/L (established), and >134 mEq/L (ART). Overall, desirable approximates were albumin >42 g/L, total protein <75 g/L, and sodium <137 mEq/L. We conclude that the optimal set points of the strongest CD4^+^ count clinical covariates tended to drift and adapt to either new ranges or overlapped with the known reference ranges to positively influence the CD4^+^ cell counts. Recommendation for phase-specific CD4^+^ cell count influence in adaptation to HIV invasion includes monitoring of the strongest covariates related to dietary conditions (sodium, albumin, and total protein), tissue oxygenation (red blood cells and its haematocrit), and hormonal control (LDH and ALP).

## 1. Introduction

Monitoring the health status of HIV infected patients is a quite complex process due to the dynamism surrounding the epidemic. This includes the socioeconomic variations associated with HIV patients' attitude towards adherence to health care [1, 2], the rapid mutation of the HIV [3–5], coinfections [6], and the biological complexity of the human body. The challenge is further exacerbated by the disease progression from one phase to the other over time [7, 8]. CD4^+^ count is the most common indicator for monitoring the HIV disease progression [9]. Equally important to understand is the drivers or covariates of the CD4^+^ count too. In the Northwest Ethiopia [10], some determinants of the CD4^+^ count change were found to be age, weight, baseline CD4^+^ cell count, cell phone ownership, visiting times, marital status, residence area, and level of disclosure of the disease to family members. In addition to this, Montarroyos et al. [11] found other factors such as smoking, use of illicit drugs, hospital treatment, changing doctors, and the use of ART. Contrary to some of these findings, age and gender were not associated with CD4^+^ count change according to [12] who also found white ethnicity to be a factor. In Iran [13], insurance coverage, tuberculosis prophylaxis, and a higher baseline CD4^+^ count were found to be protective factors. However, little attention has been given to the effects of the clinical attributes on the CD4^+^ count change.

As a defined approach to understand the disease, HIV/AIDS prospective cohort studies [14, 15] have in conjunction with the CD4^+^ count routinely gathered more information from different clinical platforms during the patient follow-up care. These CD4^+^ count clinical covariates usually appear in electronic health records (EHRs) and inherently contain information on the harmonious anatomical systems that are by nature self-regulatory to maintain optimal set points of all the variables needed for a healthy status. Hence, the clinical covariates effect should ideally contribute to the overall health status whilst confined to the desirable limits conducive for supporting life. The human immunodeficiency virus (HIV) is notoriously known for taking siege and attacking the immune system causing the CD4^+^ count fluctuations [8]. The self-regulatory immune system responds by attempting to restore the CD4^+^ count, but little is known about the complexity around the corresponding adaptation on the optimal set points of the many clinical attributes stored as EHR in the HIV/AIDS prospective cohort studies. Because of the catch-22 situation in the HIV treatment, where therapy is essential for viral suppression [16] but at the same time associated with life-threatening side effects [17], the nutritional value has also been recommended for managing the HIV disease [18]. As such, and following a study by [19] on the need to regulate the high serum calcium in HIV patients, we further postulated that, among the CD4^+^ count clinical covariates, there are some whose optimal set points that can be controlled by dietary conditions or otherwise to enhance the CD4^+^ count in accordance with the demands of the patient's health status at each specific phase of the HIV disease progression.

Previously suggested covariates of the CD4^+^ count from different clinical categories include the full blood count [20–26], lipids [27–29], sugar [30–32], blood chemistry [21, 33–50], and clinical examination measurements [51–60]. The volume of such information has increased tremendously in the recent years where it is now coined “big data” [61, 62]. This is owing to the new era of information technology where patient EHRs are being stored at a faster pace and relatively cheaper than in the past [63]. However, it has been reported that there exist few strongest clinical covariates of the CD4^+^ count [64]. The objective of this study is to harness these strongest covariates for they are attractive in providing a streamlined application of the multidimensional viewing lens (statistical models) to zoom into the behavioural patterns of the adaptive optimal set points. The aim is to visually explore the CD4^+^ count behaviour in response to the strongest covariates, focusing particularly on identifying the ranges within which they have desirable effects.

Given continuous covariates *x*_*k*_ for *k*=1,…, *p*, we intend to visualise generalised additive mixed model (GAMM) smooth curves of the response, CD4^+^ cell count denoted by *y* such that *y*=*s*_0_+∑_*k*=1_^*p*^*s*_*k*_(*x*_*k*_)+∑_*k*=1_^*p*^*s*_*k*_(*x*_*k*_, *z*)+*ε*, where *s*_0_ is the cohort's CD4^+^ count average (intercept), *s*_*k*_(*x*_*k*_) is the overall CD4^+^ count trend in response to the *k*^*th*^ covariate *x*_*k*_, the *s*_*k*_(*x*_*k*_, *z*) being the phase-specific (*z*) CD4^+^ count trends in response to the *k*^*th*^ covariate *x*_*k*_, and *ε* is the random error. Autocorrelation to capture the dependency relationship between the repeated measurements is also accounted for to improve the model accuracy. The vertical axis of the smoothed curves in the graphical displays show values around zero representing mean centred values of the response [65, 66]. Positive and negative smoothed values will then indicate that the original response value was above and below the average response value, respectively. If the smooth interaction factor *z*, the infection phase, has *g*=1,…, *G* levels, the smooth curves of the response (CD4^+^ count) within each level (phase) are displayed separately. The smooth complexities can be preset to *M* − 1 and *MG* for the overall and within level smooths, respectively, where *M* is called the basis dimension. Effective degrees of freedom (edf) are used to indicate the actual complexity captured by the fitted model, with edf approaching zero, suggesting insignificant effect on the response due to that particular covariate. Similarly, edf close to zero for an interaction factor reveals insignificant difference between the response curves across the factor levels. The evidence of insignificance is echoed by corresponding higher *p* values. In general, the GAMM is a powerful data-driven visualisation technique that discovers the response curve patterns that cannot easily be imagined. Furthermore, the interpretation is not based on the original response values but rather a transformed scale that provides good prediction or interpolation in exploring the functional nature of the response behaviour. For more details, the interested reader may consult [67–73] for GAMM.

## 2. Materials and Methods

### 2.1. Study Design

**Figure 1:**
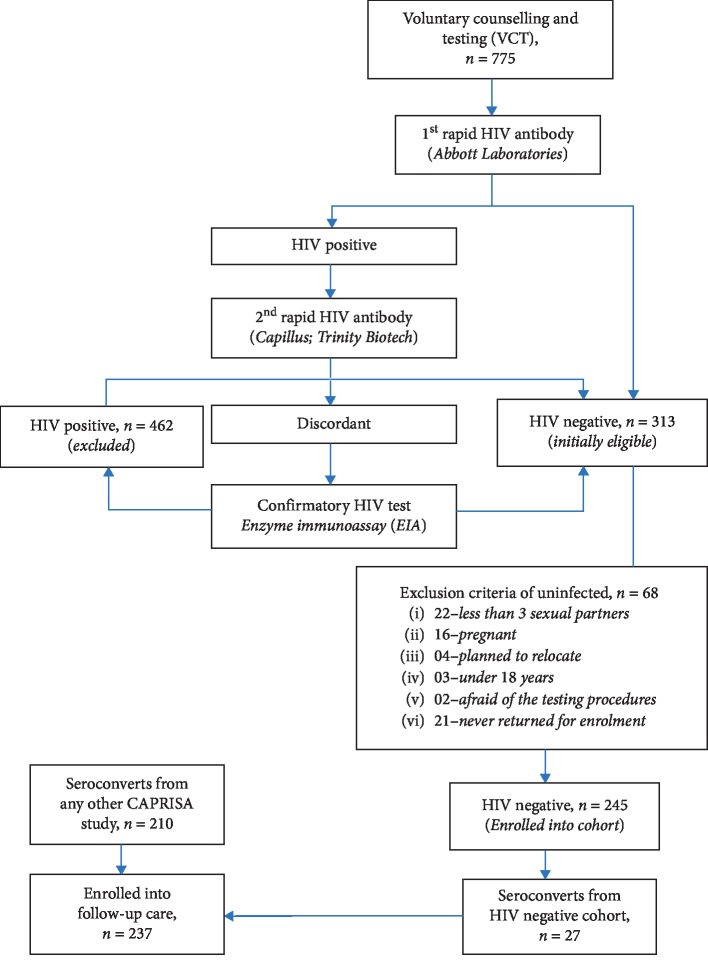
The study design. The CAPRISA 002 HIV negative cohort (phase 1: pre-HIV infection) screening involved 775 voluntary potential candidates of which 462 were already HIV positive and 313 initially eligible. Of the 313 HIV negative, only 245 were enrolled and the rest were excluded for various reasons according to the eligibility criteria. Eventually, 27 out of the 245 seroconverted and enrolled into follow-up care. Seroconverts from other CAPRISA studies (210) were also included into the follow-up care that resulted in a total of 237 patients for this study.

Figure 1 summarises the study design at the Centre for the AIDS Programme of Research in South Africa (CAPRISA), where a total sample size of 237 seroconverts whose records were investigated. The establishment of the acute infection study for the female sex workers, cohort screening and seroconverts, routine evaluation procedures, CAPRISA-participant interaction, and data management have been previously documented [74]. The study protocol and informed consent documents were reviewed and approved by the local ethics committees of the University of KwaZulu-Natal, the University of Cape Town, the University of the Witwatersrand in Johannesburg, and by the Prevention Sciences Review Committee (PSRC) of the Division of AIDS (DAIDS, National Institutes of Health, USA). The consent forms were translated into vernacular language, isiZulu, and written informed consent was obtained at each stage of the study. All the minors, under the age of 18 years, were excluded from the study as part of the screening procedure. The HIV negative cohort (phase 1: pre-HIV infection) was followed up, and upon HIV infection, they were further followed up weekly to fortnightly visits up to 3 months (phase 2: acute infection), monthly visits from 3 to 12 months (phase 3: early infection), quarterly visits, and thereafter (phase 4: established infection) until ART initiation (phase 5). Eventually, 27 seroconversions were recorded. In addition to the 27 seroconverts, 210 more patients who seroconverted from other CAPRISA studies were also enrolled and similarly followed up postinfection from acute to the ART phase. This study investigated the repeated measurements obtained from all the 237 patients.

### 2.2. Data

Four time points prior to each phase transition were selected giving rise to a total of 16 repeated measurements being investigated for each patient. The baseline (Phase 1) repeated measurements were scarce, and hence, this study focused on phases 2 to 5 only. The strongest CD4^+^ count clinical covariates reported in a previous study [64] are shown in Table 1 and were mostly clinical attributes from laboratory tests.

### 2.3. Statistical Analysis

The analysis was performed in the open source R software, version 3.5.0 of the R Core Team. The function bam (for large datasets) was used for fitting the GAMM with a factor smooth interactions basis fs in the library *mgcv* whilst incorporating an AR(1) structure using the library *itsadug*. The random smooths for the interaction factor *z*, which is the infection phase, had levels *g*=1 (phase 2: acute), *g*=2 (phase 3: early), *g*=3 (phase 4: Est), and *g*=4 (phase 5: ART). Hence, *G*=4. The basis dimension was set to *M*=5 assuming that the curvatures were slightly complex than cubic splines. Note that the bam syntax in R software uses *k* ≡ *M* in this case. The random smooths were explored using the function inspect_random and complex optimal set points visualized using the vis.gam function.

## 3. Results

**Figure 2:**
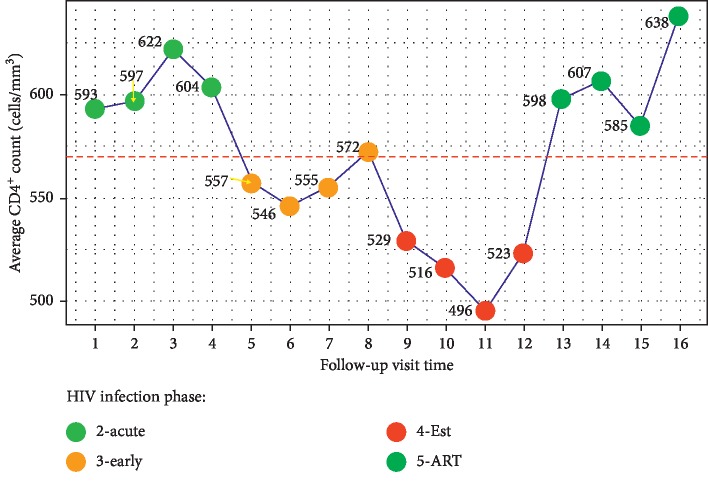
The cohort's average CD4^+^ count at each visit time. The horizontal broken line represents the cohort's average CD4^+^ count of 571 cells/mm^3^ for the entire follow-up period.

Figure 2 shows the cohort's average CD4^+^ counts during the follow-up visit times. The average CD4^+^ counts at the visit time points during the acute and ART phases were above the cohort's average of 571 cells/mm^3^, whilst the early and established phases were below the average.  Table 2 is a summary of the significance of the terms in the fitted model. The intercept was estimated to be *s*_0_ = 574.3461 cells/mm^3^ with *p* < 0.001, an average value very close to the observed overall cohort average 571 cells/mm^3^ of the CD4^+^ cell counts. We then defined a covariate's optimal set point(s) as the threshold point(s) above or below which the corresponding CD4^+^ cell counts were above this average. The overall covariate smooth terms for s(MCHC) with *p*=0.3866; s(calcium) with *p*=0.7152; s(magnesium) with *p*=0.7418; and s(potassium) with *p*=0.3348 were statistically insignificant contributors to the CD4^+^ count changes. Consequently, all the corresponding CD4^+^ count trends within the separate HIV infection phases were also not significantly different from each other in response to these covariates. This was also confirmed by their smooth terms, showing very small effective degrees of freedom. The random smooth terms for s(MCV, phase) with *p*=0.3808; s(monocytes, phase) with *p*=0.2127; s(basophils, phase) with *p*=0.4206; and s(folate, phase) with *p*=0.7075 indicated that there was no sufficient evidence to suggest a significant difference in the CD4^+^ count trends between the infection phases in response to these respective clinical covariates. However, their overall smooth terms contributed to the CD4^+^ count changes during the follow-up period. The *k*-indices are close to 1, an indication that it is less likely that there were missing patterns in the residuals.

### 3.1. CD4^+^ Count Trends in response to the Strongest Clinical Covariates and Their Optimal Set Points

#### 3.1.1. Significant Difference between the Random Smooths

**Figure 3:**
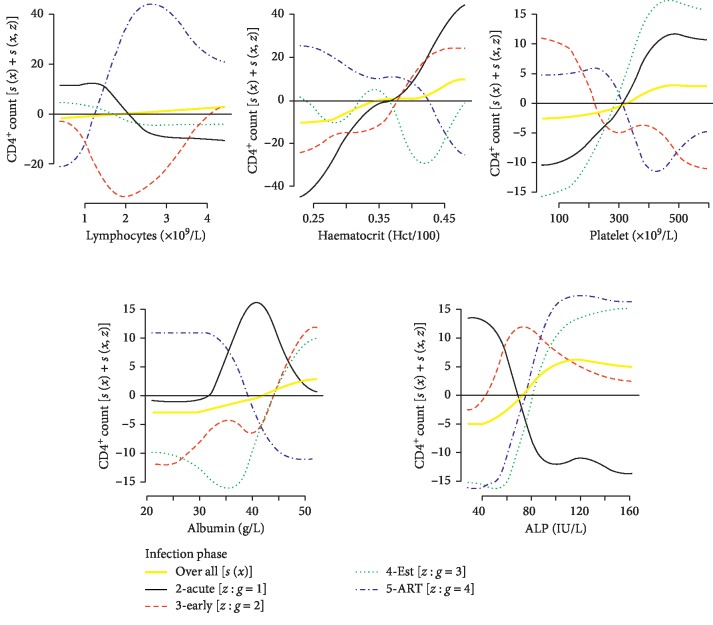
Significant difference between random smooths and overall upward trends.

*(1) Overall Upward Trends*.  Figure 3 shows the covariates that positively influenced the CD4^+^ cell count overall and having different trends within the HIV infection phases. Generally, an increase in lymphocytes, haematocrit, platelets, albumin, and ALP was associated with an improved CD4^+^ cell count. With the exception of ALP, they showed an almost overall direct relationship with the CD4^+^ count although the rates of change were fairly low. An increase in ALP approx. between 60 and 100 IU/L resulted in a sharp increase in the overall CD4^+^ count and then levelled off thereafter. The overall upward CD4^+^ count trends exceeded the cohort's average at approx. lymphocytes count >2 × 10^9^/L, haematocrit >35%, platelet count >350 × 10^9^/L, albumin >42 g/L, and ALP >70 IU/L.

The behavioural patterns of the random smooths for the general upward trends were quite complex. Recalling that the average observed CD4^+^ counts for the early and established phases were below the cohort's average (Figure 2), the GAMM plot showed that, during the early phase, the CD4^+^ count remained below the cohort average despite the increase in the lymphocytes. During the acute and established phases, the CD4^+^ count declined in response to the lymphocytes increase in the range approx. <2 × 10^9^/L. At lymphocytes count approx. <2.5 × 10^9^/L, ART supported a direct influence on the CD4^+^ count and this relationship diminished as lymphocytes increased beyond the 2.5 × 10^9^/L, but the CD4^+^ cell counts remained well above average. Above this point (approx. 2.5 × 10^9^/L), the CD4^+^ cell counts in the pretreatment phases were below average. In response to haematocrit, the CD4^+^ cell count was staggering below the average during the established phase, the period during which the lowest CD4^+^ counts were recorded. The CD4^+^ counts increased with increase in the haematocrit during the acute and early phases whilst declining during medication (ART). The interaction with medication showed that the CD4^+^ count dropped to below average at haematocrit approx. >40%. Since the CD4^+^ count was negatively related to the haematocrit during the ART phase and positively in both the acute and early phases, the plot indicated that maintaining the haematocrit within the neighbourhood of approx. 40% improved the CD4^+^ count to above average in all the three phases (acute, early, and ART). According to our data recorded at the lowest levels of CD4^+^ counts (established phase) and during high viral load (acute phase), an increase in the platelet count positively influenced the CD4^+^ counts. Desirable linear effects were at platelet count approx. >275 × 10^9^/L and levelled off at approx. >450 × 10^9^/L. The trends showed that the rate of such platelet influence was higher during the established phase than the acute phase. These trends were opposite to those observed in the early and ART phases where the influence on the CD4^+^ cell count to above average was only at a lower platelet count approx. <200 × 10^9^/L. During the acute phase, desirable CD4^+^ counts were observed at almost the entire range of the recorded albumin measurements (20–50 g/L) with the most desirable effects in the neighbourhood of approx. 40 g/L. The ART trend behaved oppositely to those of both the early and established phases in response to the albumin. During the ART, the albumin desirably influenced the CD4^+^ cell count at lower levels of approx. <37 g/L, yet the early and the established phases required that the albumin be approx. >45 g/L. The random smooths shapes of the ALP effect on the CD4^+^ count were almost the same during the established and ART phases with the ART, showing a slightly better influence on the CD4^+^ cell count. With the exception of the acute phase, all the other infection phases showed that, at ALP approx. >80 IU/L, the CD4^+^ count cell count was above average. The early phase had desirable effects during the entire range of the recorded measurements (40–160 IU/L). The ALP and CD4^+^ cell counts were inversely related during the acute phase with the desirable effects of ALP at approx. <60 IU/L.

**Figure 4:**
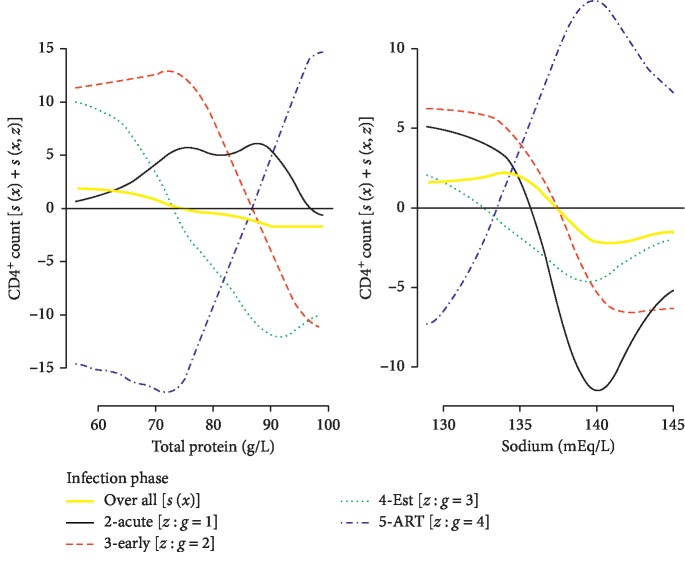
Significant difference between random smooths and overall downward trends.

*(2) Overall Downward Trends*. Generally, the cohort's total protein and sodium were negatively related to the CD4^+^ cell count with overall favourable levels approx. <75 g/L and <137 mEq/L, respectively (Figure 4). The interaction between HIV treatment and these covariates showed a positive influence on the CD4^+^ count, and the most desirable effects were observed at total protein approx. >85 g/L and sodium approx. >134 mEq/L. Although elevated sodium levels influenced CD4^+^ count to above average, the trend nosed down at approx. >140 mEq/L during this period of medication uptake. However, the CD4^+^ counts remained high above average at that sodium level approx. >140 mEq/L. During the acute phase, the CD4^+^ count remained above average in response to all the recorded total protein levels (60–100 g/L). However, at lower CD4^+^ counts (early and established phases), an increase in the protein levels negatively impacted on the CD4^+^ count with the lowest CD4^+^ counts (established phase) being the hard hit. Desirable effects of the total protein on the CD4^+^ count were observed at approx. <75 g/L during the established phase, whereas at approx. <85 g/L, in the early phase. The sodium had negative effects on the CD4^+^ count during all the pretreatment phases with the established phase being the most affected again. The plot indicated that all the pretreatment phases would generally influence the CD4^+^ cell count to above average at optimally lower sodium levels of approx. <135 mEq/L with more restricted desirable effects during the established phase (approx. <132 mEq/L).

**Figure 5:**
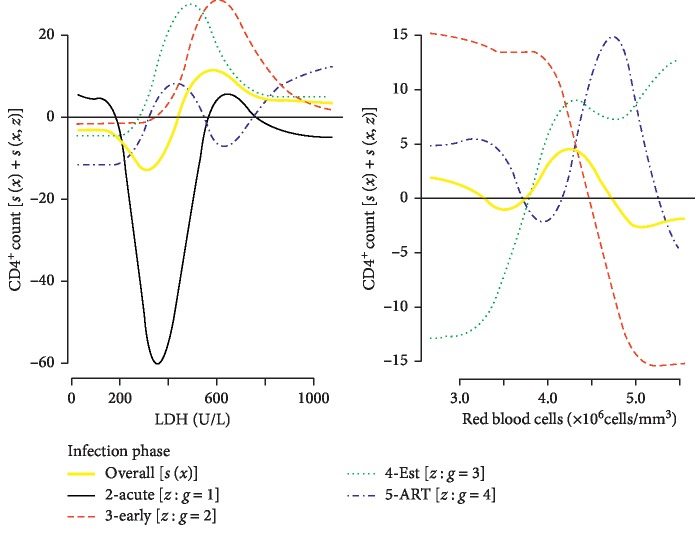
Significant difference between random smooths and overall irregular trends.

*(3) Irregular Trends (more complex)*. An increase in the LDH and red blood cells produced complex trends in both the overall and the within phase CD4^+^ count trends (Figure 5). Although fluctuations existed in the overall CD4^+^ count trend in response to LDH, the CD4^+^ count remained fairly constant and above average at approx. >500 U/L of LDH. On the contrary, the overall CD4^+^ count trend in response to the red blood cells fluctuated around the mean. The effects of medication were associated with CD4^+^ count trends that also fluctuated in response to both the LDH and red blood cells. Both covariates were hardly associated with CD4^+^ counts above average during the acute phase. At lower records of the CD4^+^ counts (early and established phases), the LDH of approx. >300 U/L showed desirable effects on the CD4^+^ count. In response to the red blood cells during these early and established phases, only the lowest records of the CD4^+^ counts (established phase) positively responded to the red blood cell increase. The plot revealed that the optimal red blood cell count for both the early and established phases could be set in the neighbourhood of approx. 4.2 × 10^6^ cells/mm^3^.

#### 3.1.2. Insignificant Differences between the Random Smooths

**Figure 6:**
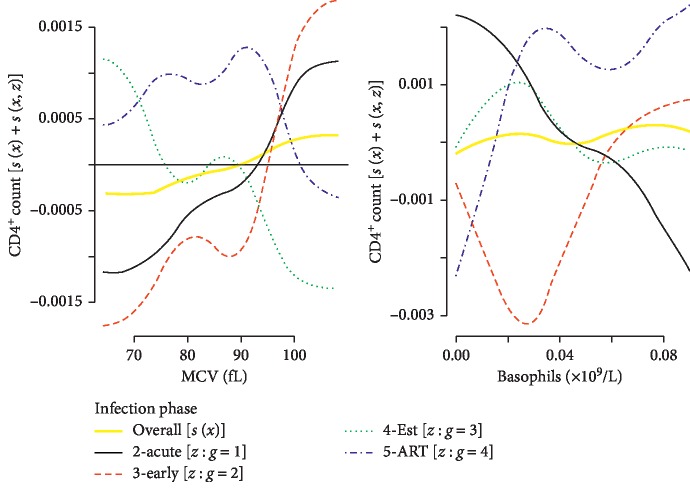
Insignificant difference between random smooths and overall upward trend.

*(1) Overall Upward Trends*. Although the random smooths for MCV and basophils showed different shapes (Figure 6), these trends were found to be statistically and insignificantly different. However, the overall CD4^+^ count trends showed a statistically significant increase in response to unit increase in these covariates. The plot showed that the cohort's overall MCV supported the CD4^+^ count to be above average at approx. >90 fL. Generally, the increase in the basophils corresponded to an increase in the CD4^+^ count but fluctuating very closely to the cohort's average.

**Figure 7:**
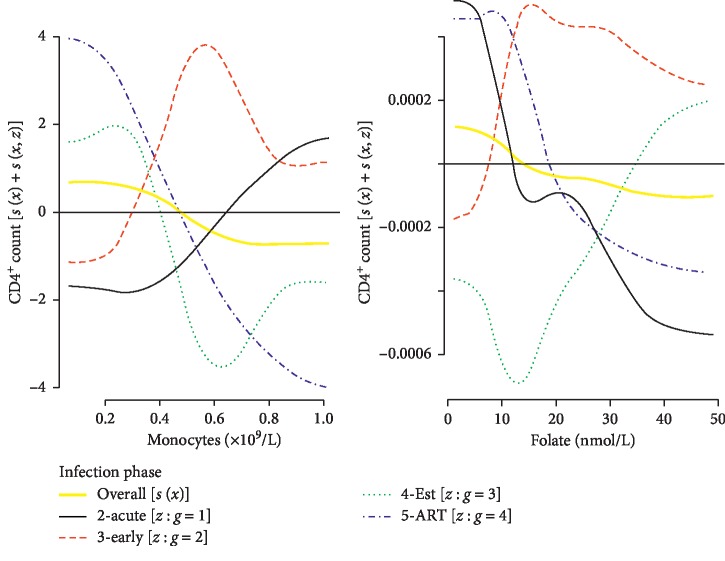
Insignificant difference between random smooths and overall downward trend.

*(2) Overall Downward Trends*. Similarly, there was no significant difference in the effect of monocytes and folate on the CD4^+^ count across the HIV infection phases (Figure 7). However, the general increase in these covariates was associated with a significant decline in the CD4^+^ cell count. The overall trends indicated that the monocytes count and folate showed desirable effects on the CD4^+^ cell count at measurements of approx. <0.5 × 10^9^/L and <15 nmol/L, respectively.

Despite the different shapes of the CD4^+^ count trends either overall or within the infection phases, potassium, magnesium, calcium, and MCHC had no effect on the CD4^+^ count behavioural changes ([Supplementary-material supplementary-material-1]).

## 4. Discussion

This study visually examined the CD4^+^ count trends in response to the strongest clinical covariates in an attempt to discover possible covariate adaptive optimal set points for positively influencing the CD4^+^ cell count in HIV infected patients. Among the strongest CD4^+^ count covariates are the lymphocytes that are B or T cells [25, 75, 76], which also consists of the CD4^+^ cells, a T cell type [77]. Hence, we found the overall linear relationship between the lymphocytes and CD4^+^ count. Since the HIV is known to mainly attack the CD4^+^ cells [8], this suggests the decline in the CD4^+^ count during the pretreatment phases of our data despite the lymphocytes increase. The suppression of HIV during the ART [16] had consequently seen the high number of CD4^+^ cells being spared during this treatment phase. These findings on the CD4^+^ count behaviour in response to lymphocytes were a confirmation of the expected results giving confidence on the accuracy of the fitted model. Monocytes for fighting against pathogens [76] have been reported to be infected by HIV [78] such that their count was supposed to be similarly affected as the CD4^+^ count. However, we observed a paradox in our data where there was an overall inverse relationship between the monocytes and the CD4^+^ cell count. The damage to body tissues and inflammation as indicated by basophils [76] was only observed from an overall point and likely due to the basophils being the least abundant leucocytes [79]. A study by [80] found that a low blood clotting condition (platelet count [76, 81–83]) was associated with a low CD4^+^ count. Our data confirmed the same relationship but holding only during the period of high viral load (acute [84, 85]) and established phases where the lowest CD4^+^ counts were recorded. During these two phases, the optimal set point for the platelet count was observed to be approx. >450 × 10^9^/L, which was higher than the normal reference range of 178–454 × 10^9^/L [86, 87].

The general increase in the CD4^+^ cells in response to the tissue oxygenation, based on haematocrit and MCV, was also observed in a study by Vanisri and Vadiraja [20]. This is likely because these two clinical covariates are both red blood cell indices for determining the level of tissue oxygenation [88–90]. The indices' contribution to the CD4^+^ count was greatly affected during high viral load the acute phase. The red blood cells are responsible for the oxygen transportation [88, 89], and LDH catalyses the compensation of energy levels during insufficient oxygen [91]. Both were associated with lower than average CD4^+^ counts during the acute phase. This high viral replication phase [8] has been reported to have complex relationships with oxygen effects [92], which may also suggest the twisted CD4^+^ count trends in response to the LDH and red blood cell in our data. Aerobic endurance is referred to as the functional state of the oxygen transport system [93] and has been reported to be reduced in HIV positive patients than negative ones [94–96]. Our results based on the LDH suggested that aerobic endurance was associated with a negative impact on the CD4^+^ count mostly during the acute phase.

The acid-base and normal water balance (total protein [97]) supported CD4^+^ cell counts above average at high viral loads for almost all the recorded measurements of the total protein between 60 and 100 g/L. The normal total protein range is known to be between 60 and 80 g/L [98] and corresponded to the range in which our results indicated CD4^+^ count above average for the early and established phases. Also revealed in our data is that the longer the patient has been leaving with the virus without medication, the less responsive was the CD4^+^ cell count to protein levels. However, the same data showed that, during treatment, the normal protein range had negative effects on the CD4^+^ cell count. At total protein levels approx. >75 g/L during ART, a positive linear relationship with CD4^+^ count was observed and the CD4^+^ counts exceeded the average at approx. >90 g/L of total protein. This confirmed the report by [99] that the serum protein increases with highly active antiretroviral therapy which also enhances the CD4^+^ cell count [16]. Albumin which is also a type of protein [100] helps with tissue nourishment [81]. Both total protein and albumin results were consistent in positively influencing the CD4^+^ count to above average at almost all their measurements during the acute phase. The albumin normal reference range is considered to be between 35 and 50 g/L [101] and was associated with desirable CD4^+^ counts at elevated viral load in our data. However, this range corresponded to a sharp decline in the CD4^+^ count during medication. It has been reported that serum albumin concentrations increase significantly on ART initiation [102]. To positively influence the CD4^+^ cell count in response to albumin during ART, the data suggested that albumin levels be lower than normal (approx. <35 g/L) whilst higher albumin levels (approx. >45 g/L) being favourable for the early and established phases. The general direct positive relationship between albumin and the CD4^+^ count concurred with the studies in [40, 41].

The normal ALP is known to be in the range of 30–120 IU/L [103, 104], the range in which our data showed an inverse relationship with the CD4^+^ count in the presence of a high viral load (acute phase). During the acute phase, CD4^+^ cell count improved to above average at lower ALP (approx. <70 IU/L). After the acute phase, the immune system is known to fight back to restore the CD4^+^ count [85]. Within 3–12 months of infection (early phase), the CD4^+^ cell count responded well to normal ALP and remained above average. The results further showed that, as the immune system continued to fight back with (ART phase) or without treatment (established phase), the ALP showed a strong positive linear relationship with the CD4^+^ cell count. From at least 3 months of infection, the ALP's positive linear association with the CD4^+^ count diminishes beyond the normal ALP upper limit of 120 IU/L but still supporting the CD4^+^ count to above average. However, at such elevated ALP levels, it is known to be an indication of liver damage [78]. Sodium also like calcium plays a crucial role in the regulation of water balance, blood pressure, blood volume, heart rhythm, and the brain and nerve function [76, 81, 105]. Under normal circumstances, it operates between 135 and 145 mEq/L [106, 107]. The results also indicated that there was a shift in the sodium optimal range where measurements below the normal range during the pretreatment phases were associated with an improved CD4^+^ count above average. This may suggest the changes in the osmotic gradient between extracellular and intracellular fluid in cells due to sodium [108] in the presence of viral infection before treatment. Upon viral suppression during ART, there was a direct relationship between the sodium and CD4^+^ count. A similar positive correlation was observed in [45] among HIV positive patients but without considering the infection phase. Our data further revealed that the positive correlation during ART tails off at approx. >140 mEq/L of sodium but still influencing the CD4^+^ count to reach levels above average. Many foods naturally contain folate, a B-vitamin, which is needed for cell growth and metabolism [109, 110]. Contrary to [33] that it improves the CD4^+^ count, our data showed that generally a unit increase in the folate was associated with a drop in the CD4^+^ cell count.

Of the highlighted main influential covariates of the CD4^+^ cell count, the findings suggest that their incorporation into the management of the HIV disease can be in three methods including dietary conditions, tissue oxygenation, and hormonal control. Sodium and the proteins can be regulated in the patient's diet [18], whereas aerobic endurance of the red blood cells requires improved physical fitness [94] in conjunction with the monitoring of the hormone LDH. The hormone ALP can possibly be administered to patients [111]. However, the actual adherence to the set points during patient care opens channels to other areas of exploration for effective implementation.

As much as our model was effective in the pattern discovery, we acknowledge the limitations of our data. The baseline records before HIV infection were not available hampering the opportunity to compare the CD4^+^ count behavioural trends and optimal set points before and after the HIV infection of the same individuals despite the availability of known reference ranges. In addition, information on the presence of other infections, comorbidities, or patients' dietary patterns including dehydration was not available, which may have acted as confounders. The study design and data collection for our investigation was done more than a decade before this analysis [74]. At that time, almost all the subjects initiated ART nearly a year after the diagnosis of the HIV, which as per present recommendations should be started as soon as diagnosis is made, provided there are no contraindications [112, 113]. This early therapy and the response to it may alter the covariates of CD4^+^ count. As such, future studies are recommended to investigate the strongest covariates and their adaptive optimal set points on data that take into account the context of the recent policies on ART initiation upon diagnosis. Furthermore, given the complexity of the course of the HIV infection, the availability of a much larger sample size is encouraged to improve the representations of the divergent presentations we have demonstrated.

## 5. Conclusions

We conclude that the optimal set points of the few strongest CD4^+^ count clinical covariates tended to drift and adapt to either new ranges or overlapped with the known reference ranges to positively influence the CD4^+^ cell counts. Recommendation for phase-specific CD4^+^ cell count influence in adaptation to HIV invasion include monitoring of the strongest covariates related to dietary conditions (sodium, albumin, and total protein), tissue oxygenation (red blood cells and its haematocrit), and hormonal control (LDH and ALP).

## Figures and Tables

**Table 1:** The previously reported strongest clinical covariates of the CD4^+^ cell count.

Response		
Health indicator	CD4^+^ cell count	Cells/mm^3^

Blood count		

Red blood cells	Red blood cells	×10^6^ cells/mm^3^
	Haematocrit	Hct/100
	MCV	fL
	MCHC	g/dL

Clotting condition	Platelets	×10^9^/L

White blood cells	Lymphocytes	×10^9^/L
	Monocytes	×10^9^/L
	Basophils	×10^9^/L

Blood chemistry		

Liver function	ALP	IU/L
Electrolytes	Calcium	mmol/L
	Magnesium	mmol/L
	Potassium	mmol/L
	Sodium	mEq/L

Protein	Total protein	g/L
	Albumin	g/L
	LDH	U/L

Vitamins	Folate	nmol/L

*Abbreviations.* MCV, mean corpuscular volume; MCHC, mean corpuscular haemoglobin concentration; ALP, alkaline phosphatase; LDH, lactate dehydrogenase. Source: [64].

**Table 2:** Significance of the CD4^+^ count clinical covariates in the fitted GAMM.

	Intercept, *s*_0_		Estimate	Std. error	*t* value	Pr (>|*t*|)
			574.3461	23.6848	24.2496	<0.001
	Smoothed trends					
*k*	*Overall, s*_*k*_(*x*_*k*_)	M-1	M-index	edf	*F*	*p* value
1	s(red blood cells)	4	0.97	0.0005	0.0001	0.2407
2	s(haematocrit)	4	0.88	2.3867	1.4682	**0.0002**
3	s(MCV)	4	0.98	3.4599	10.9677	**0.0001**
4	s(MCHC)	4	0.96	0.0005	0.0001	0.3866
5	s(platelet)	4	0.99	3.1121	7.0860	**0.0001**
6	s(lymphocytes)	4	0.98	3.9278	89.0726	**0.0001**
7	s(monocytes)	4	0.98	2.8098	6.8695	**0.0001**
8	s(basophils)	4	0.80	3.2409	5.1025	**0.0001**
9	s(ALP)	4	0.95	1.8215	1.2221	**0.0010**
10	s(calcium)	4	0.83	0.0002	0.0000	0.7152
11	s(magnesium)	4	0.80	0.0001	0.0000	0.7418
12	s(potassium)	4	0.94	0.0014	0.0003	0.3348
13	s(sodium)	4	0.89	1.8233	1.4124	**0.0022**
14	s(total protein)	4	0.89	3.6558	16.7466	**0.0001**
15	s(albumin)	4	0.86	3.3584	8.5429	**0.0001**
16	s(LDH)	4	0.91	0.0056	0.0014	**0.0167**
17	s(folate)	4	0.79	3.4563	18.2167	**0.0001**

*k*	*Phase specific, s*_*k*_(*x*_*k*_, *z*)	*MG*				

1	s(red blood cells, phase)	20	0.97	5.7216	0.5062	**0.0232**
2	s(haematocrit, phase)	20	0.88	6.8664	0.8138	**0.0008**
3	s(MCV, phase)	20	0.98	0.0006	0.0000	0.3808
4	s(MCHC, phase)	20	0.96	2.7234	0.1963	0.1484
5	s(platelet, phase)	20	0.99	4.0657	0.4305	**0.0205**
6	s(lymphocytes, phase)	20	0.98	6.4017	1.1305	**0.0001**
7	s(monocytes, phase)	20	0.98	1.2916	0.0823	0.2127
8	s(basophils, phase)	20	0.80	0.0011	0.0000	0.4206
9	s(ALP, phase)	20	0.95	5.1570	0.6649	**0.0022**
10	s(calcium, phase)	20	0.83	0.9796	0.0587	0.2741
11	s(magnesium, phase)	20	0.80	0.0011	0.0001	0.4274
12	s(potassium, phase)	20	0.94	0.0003	0.0000	0.6605
13	s(sodium, phase)	20	0.89	3.0043	0.2508	**0.0652** ^**†**^
14	s(total protein, phase)	20	0.89	3.9438	0.4838	**0.0099**
15	s(albumin, phase)	20	0.86	4.0936	0.3857	**0.0381**
16	s(LDH, phase)	20	0.91	10.1688	1.6168	**0.0001**
17	s(folate, phase)	20	0.79	0.0003	0.0000	0.7075

Bold represents significance at 5% level. ^†^Significant at 10% level.

## Data Availability

The data used to support the findings of this study are available from the corresponding author upon request.

## Supplementary Materials

Supplementary Materials File S1: the raw data for the study. The data consist of 237 patients, who were followed up through four phases (2 to 5) post-HIV infection (excluding phase 1: HIV negative), where phase 2: acute infection is basically visits up to 3 months, monthly visits from 3 to 12 months (phase 3: early infection), quarterly visits thereafter (phase 4: established infection), and until ART initiation (phase 5). The observations are the repeated measurements of CD4^+^ count and 46 clinical covariates recorded during the last four visits of each phase. Figure S1: the contour plots of the complex optimal set points. The regions in peach correspond to desirable set points, whilst the blue regions represent the undesirable range of the clinical covariates. Figure S2: insignificant terms. Changes in these measurements does not have an influence on the CD4^+^ count.

## References

[1] Burch L. S., Smith C. J., Anderson J. (2016). Socioeconomic status and treatment outcomes for individuals with HIV on antiretroviral treatment in the UK: cross-sectional and longitudinal analyses. *The Lancet Public Health*.

[2] Bunyasi E. W., Coetzee D. J. (2017). Relationship between socioeconomic status and HIV infection: findings from a survey in the free state and western cape provinces of South Africa. *BMJ Open*.

[3] Burke D. (1997). Recombination in HIV: an important viral evolutionary strategy. *Emerging Infectious Diseases*.

[4] Shafer R. W., Rhee S.-Y., Pillay D. (2007). HIV-1 protease and reverse transcriptase mutations for drug resistance surveillance. *AIDS*.

[5] Cuevas J. M., Geller R., Garijo R., López-Aldeguer J., Sanjuán R. (2015). Extremely high mutation rate of HIV-1 in vivo. *PLoS Biology*.

[6] The Lancet Infectious Diseases (2013). Co-infection: new battlegrounds in HIV/AIDS. *The Lancet Infectious Diseases*.

[7] Brener J., Gall A., Hurst J. (2018). Rapid HIV disease progression following superinfection in an HLA-B∗27:05/B∗57:01-positive transmission recipient. *Retrovirology*.

[8] Weston R., Marett B. (2009). HIV infection pathology and disease progression. *Clinical Pharmacist*.

[9] Beare A., Stockinger H., Zola H., Nicholson I. (2008). The CD system of leukocyte surface molecules: monoclonal antibodies to human cell surface antigens. *Current Protocols in Immunology*.

[10] Seyoum A., Ndlovu P., Zewotir T. (2017). Joint longitudinal data analysis in detecting determinants of CD4 cell count change and adherence to highly active antiretroviral therapy at Felege Hiwot teaching and specialized hospital, north-west Ethiopia (Amhara region). *AIDS Research and Therapy*.

[11] Montarroyos U. R., Miranda-Filho D. B., César C. C. (2014). Factors related to changes in CD4^+^ T-cell counts over time in patients living with HIV/AIDS: a multilevel analysis. *PLoS One*.

[12] Smith C. J., Sabin C. A., Youle M. S. (2004). Factors influencing increases in CD4 cell counts of HIV‐positive persons receiving long‐term highly active antiretroviral therapy. *The Journal of Infectious Diseases*.

[13] Abbastabar H., Rezaianzadeh A., Rajaeefard A., Ghaem H., Motamedifar M., Kazeroon P. A. (2016). Determining factors of CD4 cell count in HIV patients: in a historical cohort study. *International Journal of Life Science and Pharma Research*.

[14] Lorente N., Fernàndez-López L., Fuertes R. (2016). COBA-cohort: a prospective cohort of HIV-negative men who have sex with men, attending community-based HIV testing services in five European countries (a study protocol). *BMJ Open*.

[15] Phair J. P. (2009). Variations in the natural history of HIV infection. *AIDS Research and Human Retroviruses*.

[16] Hunt P. W., Deeks S. G., Rodriguez B. (2003). Continued CD4 cell count increases in HIV-infected adults experiencing 4 years of viral suppression on antiretroviral therapy. *AIDS*.

[17] Lands L., Pustil R. (2006). A practical guide to HIV drug side effects for people living with HIV/AIDS. *The Canadian AIDS Treatment Information Exchange*.

[18] Johansen D., McLay D., Thaczuk D., Nambiar D. (2007). A practical guide to nutrition for people living with HIV. *Canada: Canadian AIDS Treament Information Exchange*.

[19] Shadrack B. E., Digban K. A., Ishiaku A. A., Olayinka D., Nguku P., Bright L. E. (2014). Serum calcium level in HIV patients at federal medical center Yenagoa, Bayelsa state, Nigeria. *International Journal of Science and Research (IJSR)*.

[20] Vanisri H., Vadiraja N. (2016). Association between red blood cell parameters and immune status in HIV infected males. *Indian Journal of Pathology and Oncology*.

[21] Obirikorang C., Yeboah F. A. (2009). Blood haemoglobin measurement as a predictive indicator for the progression of HIV/AIDS in resource-limited setting. *Journal of Biomedical Science*.

[22] Vanisri H., Vadiraja N. (2016). Relationship between red blood cell parameters and immune status in HIV infected females. *Indian Journal of Pathology and Oncology*.

[23] Leticia O. I., Ugochukwu A., Ifeanyi O. E., Andrew A., Ifeoma U. E. (2014). The correlation of values of CD4 count, platelet, Pt, aptt, fibrinogen and factor VIII concentrations among HIV positive patients in FMC Owerri. *IOSR Journal of Dental and Medical Sciences*.

[24] Alavi S. M., Ahmadi F., Farhad M. (2009). Correlation between total lymphocyte count, hemoglobin, hematocrit and CD4 count in HIV/AIDS patients. *Acta Medica Iranica*.

[25] Shapiro N. I., Karras D. J., Leech S. H., Heilpern K. L. (1998). Absolute lymphocyte count as a predictor of CD4 count. *Annals of Emergency Medicine*.

[26] Sivaram M., White A., Radcliffe K. W. (2012). Eosinophilia: clinical significance in HIV-infected individuals. *International Journal of STD & AIDS*.

[27] Iffen T. S., Efobi H., Usoro C. A. O., Udonwa N. E. (2010). Lipid profile of HIV-positive patients attending university of calabar teaching hospital, Calabar-Nigeria. *World Journal of Medical Sciences*.

[28] Oka F., Naito T., Oike M. (2012). Correlation between HIV disease and lipid metabolism in antiretroviral-naı¨ve HIV-infected patients in Japan. *Journal of Infection and Chemotherapy*.

[29] Floris-Moore M., Howard A., Lo Y., Arnsten J. H., Santoro N., Schoenbaum E. E. (2006). Increased serum lipids are associated with higher CD4 lymphocyte count in HIV-infected women. *HIV Medicine*.

[30] Misra R., Chandra P., Riechman S. E. (2013). Relationship of ethnicity and CD4 Count with glucose metabolism among HIV patients on highly-active antiretroviral therapy (HAART). *BMC Endocrine Disorders*.

[31] Maganga E., Smart L. R., Kalluvya S. (2015). Glucose metabolism disorders, HIV and antiretroviral therapy among Tanzanian adults. *PLoS One*.

[32] McKnight T. R., Yoshihara H. A. I., Sitole L. J. (2014). A combined chemometric and quantitative NMR analysis of HIV/AIDS serum discloses metabolic alterations associated with disease status. *Molecular BioSystems*.

[33] Adhikari P. M., Chowta M. N., Ramapuram J. T., Rao S. B., Udupa K., Acharya S. D. (2016). Effect of vitamin B12 and folic acid supplementation on neuropsychiatric symptoms and immune response in HIV-positive patients. *Journal of Neurosciences in Rural Practice*.

[34] Semeere A. S., Nakanjako D., Ddungu H., Kambugu A., Manabe Y. C., Colebunders R. (2012). Sub-optimal vitamin B-12 levels among ART-naı¨ve HIV-positive individuals in an urban cohort in Uganda. *PLoS One*.

[35] Volberding P. A., Levine A. M., Dieterich D. (2004). Anemia in HIV infection: clinical impact and evidence-based management strategies. *Clinical Infectious Diseases*.

[36] Butt A. A., Michaels S., Greer D., Clark R., Kissinger P., Martin D. H. (2002). Serum LDH level as a clue to the diagnosis of histoplasmosis. *The AIDS Reader*.

[37] Butt A. A., Michaels S., Kissinger P. (2002). The association of serum lactate dehydrogenase level with selected opportunistic infections and HIV progression. *International Journal of Infectious Diseases*.

[38] Sudfeld C. R., Isanaka S., Aboud S. (2013). Association of serum albumin concentration with mortality, morbidity, CD4 T-cell reconstitution among Tanzanians initiating antiretroviral therapy. *Journal of Infectious Diseases*.

[39] Moolla Y., Moolla Z., Reddy T., Magula N. (2015). The use of readily available biomarkers to predict CD4 cell counts in HIV-infected individuals. *South African Family Practice*.

[40] Santos A. C. O. D., Almeida A. M. R. (2013). Nutritional status and CD4 cell counts in patients with HIV/AIDS receiving antiretroviral therapy. *Revista da Sociedade Brasileira de Medicina Tropical*.

[41] Pralhadrao H. S., Kant C., Phepale K., Mali M. K. (2016). Role of serum albumin level compared to CD4^+^ cell count as a marker of immunosuppression in HIV infection. *Indian Journal of Basic and Applied Medical Research*.

[42] Voss T. G., Fermin C. D., Levy J. A., Vigh S., Choi B., Garry R. F. (1996). Alteration of intracellular potassium and sodium concentrations correlates with induction of cytopathic effects by human immunodeficiency virus. *Journal of Virology*.

[43] Choi B., Gatti P. J., Haislip A. M., Fermin C. D., Garry R. F. (1998). Role of potassium in human immunodeficiency virus production and cytopathic effects. *Virology*.

[44] Khaidukov S. V., Litvinov I. S. (2005). Calcium homeostasis change in CD4^+^ T lymphocytes from human peripheral blood during differentiation in vivo. *Biochemistry (Moscow)*.

[45] Braconnier P., Delforge M., Garjau M., Wissing K. M., De Wit S. (2017). Hyponatremia is a marker of disease severity in HIV-infected patients: a retrospective cohort study. *BMC Infectious Diseases*.

[46] Bani-Sadr F., Lapidus N., Rosenthal E. (2009). Gamma glutamyl transferase elevation in HIV/hepatitis C virus–coinfected patients during interferon–ribavirin combination therapy. *JAIDS Journal of Acquired Immune Deficiency Syndromes*.

[47] Fleischbeina E., O’Brienb J., Martelinoc R., Fenstersheibd M. (2008). Elevated alkaline phosphatase with raltegravir in a treatment experienced HIV patient. *AIDS*.

[48] Gomo E., Ndhlovu P., Vennervald B., Nyazema N., Friis H. (2001). Enumeration of CD4 and CD8 T-cells in HIV infection in Zimbabwe using a manual immunocytochemical method. *Central African Journal of Medicine*.

[49] Dusingize J. C., Hoover D. R., Shi Q. (2015). Association of abnormal liver function parameters with HIV serostatus and CD4 count in antiretroviral-naive Rwandan women. *AIDS Research and Human Retroviruses*.

[50] Shiferaw M. B., Tulu K. T., Zegeye A. M., Wubante A. A. (2016). Liver enzymes abnormalities among highly active antiretroviral therapy experienced and HAART Na\ve HIV-1 infected patients at debre tabor hospital, NorthWest Ethiopia: a comparative cross-sectional study. *AIDS Research and Treatment*.

[51] Dannhauser A., van Staden A., van der Ryst E. (1999). Nutritional status of HIV-1 seropositive patients in the free state province of South Africa: anthropometric and dietary profile. *European Journal of Clinical Nutrition*.

[52] Dimala C. A., Kadia B. M., Kemah B.-L., Tindong M., Choukem S.-P. (2018). Association between CD4 cell count and blood pressure and its variation with body mass index categories in HIV-infected patients. *International Journal of Hypertension*.

[53] Nzou C., Kambarami R. A., Onyango F. E., Ndhlovu C. E., Chikwasha V. (2010). Clinical predictors of low CD4 count among HIV-infected pulmonary tuberculosis clients: a health facility-based survey. *South African Medical Journal*.

[54] Kwantwi L. B., Tunu B. K., Boateng D., Quansah D. Y. (2017). Body mass index, haemoglobin, and total lymphocyte count as a surrogate for CD4 count in resource limited settings. *Journal of Biomarkers*.

[55] Esposito F. M., Coutsoudis A., Visser J., Kindra G. (2008). Changes in body composition and other anthropometric measures of female subjects on highly active antiretroviral therapy (HAART): a pilot study in Kwazulu-Natal, South Africa. *The Southern African Journal of HIV Medicine*.

[56] Fofana K. C. (2016). Correlation between nutritional indicators and low CD4 count (200 cells·mm^3^) among HIV positive adults in Kapiri, Zambia.

[57] Venter E., Gericke G., Bekker P. (2009). Nutritional status, quality of life and CD4 cell count of adults living with HIV/AIDS in the Ga-Rankuwa area (South Africa). *South African Journal of Clinical Nutrition*.

[58] Manner I. W., Trøseid M., Oektedalen O., Baekken M., Os I. (2013). Low nadir CD4 cell count predicts sustained hypertension in HIV-infected individuals. *The Journal of Clinical Hypertension*.

[59] Hsue P. Y., Hunt P. W., Ho J. E. (2010). Impact of HIV infection on diastolic function and left ventricular mass. *Circulation: Heart Failure*.

[60] Palacios R., Santos J., Garcia A. (2006). Impact of highly active antiretroviral therapy on blood pressure in HIV-infected patients. A prospective study in a cohort of naive patients. *HIV Medicine*.

[61] Demchenko Y., Ngo C., Membrey P., Amsterdam U. V. (2013). *Architecture Framework and Components for the Big Data Ecosystem Draft*.

[62] Hersh W. R., Hoyt R. E., Yoshihashi A. (2014). Healthcare data analytics. *Health Informatics: Practical Guide for Healthcare and Information Technology Professionals*.

[63] Sun J., Reddy C. K. (2013). *Big Data Analytics for Healthcare*.

[64] Tinarwo P., Zewotir T., Yende-Zuma N., Garrett N. J., North D. (2019). An evaluation to determine the strongest CD4 count covariates during HIV disease progression in women in South Africa. *Infectious Diseases and Therapy*.

[65] Clark M. (2014). *Getting Started with Additive Models in R*.

[66] Fan J., Maity A., Wang Y., Wu Y. (2013). Parametrically guided generalized additive models with application to mergers and acquisitions data. *Journal of Nonparametric Statistics*.

[67] Wood S. N. (2010). *Generalized Additive Models*.

[68] Hastie T., Tibshirani R. (1986). Generalized additive models. *Statistical Science*.

[69] Wood S. N. (2017). *Generalized Additive Models: an Introduction with R*.

[70] Faraway J. (2006). *Extending the Linear Model with R. Generalised Linear, Mixed Effects and Nonparametric Regression Models*.

[71] Wood S. N. (2000). Modelling and smoothing parameter estimation with multiple quadratic penalties. *Journal of the Royal Statistical Society: Series B (Statistical Methodology)*.

[72] Yee T. W., Mitchell N. D. (1991). Generalized additive models in plant ecology. *Journal of Vegetation Science*.

[73] Xiang D. H. (2001). *P256-26 Fitting Generalized Additive Models with the GAM Procedure. Statistics, Data Analysis, and Data Mining*.

[74] van Loggerenberg F., Mlisana K., Williamson C. (2008). Establishing a cohort at high risk of HIV infection in South Africa: challenges and experiences of the CAPRISA 002 acute infection study. *PLoS One*.

[75] Obirikorang C., Quaye L., Acheampong I. (2012). Total lymphocyte count as a surrogate marker for CD4 count in resource-limited settings. *BMC Infectious Diseases*.

[76] Inform P. (2007). *Monitoring HIV Blood Work: A Complete Guide for Monitoring HIV*.

[77] Papagno L., Spina C. A., Marchant A. (2004). Immune activation and CD8+ T-cell differentiation towards senescence in HIV-1 infection. *PLoS Biology*.

[78] Pasupathi P., Bakthavathsalam G., Saravanan G., Devaraj A. (2008). Changes in CD4^+^ cell count, lipid profile and liver enzymes in HIV infection and AIDS patients. *Journal of Applied Biomedicine*.

[79] Min B., Brown M. A., LeGros G. (2011). Understanding the roles of basophils: breaking dawn. *The Journal of Cells, Molecules, Systems and Technologies*.

[80] Sloand E., Klein H., Banks S., Vareldzis B., Merritt S., Pierce P. (1992). Epidemiology of thrombocytopenia in HIV infection. *European Journal of Haematology*.

[81] James A. G., James T. (2017). Understanding blood tests. *Cancer Hospital*.

[82] National Institutes of Health Clinical Center (2015). *Understanding Your Complete Blood Count (CBC) and Common Blood Deficiencies*.

[83] NAM, CD4, Viral Load & Other Tests, UK, 2012

[84] Bellan S. E., Dushoff J., Galvani A. P., Meyers L. A. (2015). Reassessment of HIV-1 acute phase infectivity: accounting for heterogeneity and study design with simulated cohorts. *PLoS Medicine*.

[85] Manoto S. L., Lugongolo M., Govender U., Mthunzi-Kufa P. (2018). Point of care diagnostics for HIV in resource limited settings: an overview. *Medicina*.

[86] Omuse G., Maina D., Mwangi J. (2018). Complete blood count reference intervals from a healthy adult urban population in Kenya. *PLoS One*.

[87] Lawrie D., Coetzee L. M., Becker P., Mahlangu J., Stevens W., Glencross D. K. (2009). Local reference ranges for full blood count and CD4 lymphocyte count testing. *South African Medical Journal*.

[88] Jensen F. B., Fago A., Weber R. E. (1998). Hemoglobin structure and function. *Fish Physiology*.

[89] Wintrobe M., Greer J. (2009). *Wintrobe’s Clinical Hematology*.

[90] Arika W., Nyamai D., Musila M., Ngugi M., Njagi E. (2016). Hematological markers of in vivo toxicity. *Journal of Hematology & Thromboembolic Diseases*.

[91] Valvona C. J., Fillmore H. L., Nunn P. B., Pilkington G. J. (2016). The regulation and function of lactate dehydrogenase a: therapeutic potential in brain tumor. *Brain Pathology*.

[92] Morinet F., Parent M., Capron C., Pillet S., Bergeron C. (2015). Oxygen and viruses: a breathing story. *Journal of General Virology*.

[93] Baquet G., Van Praagh E., Berthoin S. (2003). Endurance training and aerobic fitness in young people. *Sports Medicine*.

[94] Chisati E., Vasseljen O. (2015). Aerobic endurance in HIV-positive young adults and HIV-negative controls in Malawi. *Malawi Medical Journal*.

[95] Oursler K. K., Sorkin J. D., Smith B. A., Katzel L. I. (2006). Reduced aerobic capacity and physical functioning in older HIV-infected men. *AIDS Research and Human Retroviruses*.

[96] Cade W. T., Fantry L. E., Nabar S. R., Keyser R. E. (2003). Decreased peak arteriovenous oxygen difference during treadmill exercise testing in individuals infected with the human immunodeficiency virus. *Archives of Physical Medicine and Rehabilitation*.

[97] Egyptian Company for Biotechnology (2007). *Total Protein: Biuret Reagent*.

[98] Busher J. T. (1990). *Clinical Methods: The History, Physical, and Laboratory Examinations*.

[99] Ugwuja E. I., Eze N. A. (2007). A comparative study of serum electrolytes, total protein, calcium and phosphate among diabetic and HIV/AIDS patients in abakaliki, southeastern, Nigeria. *The Internet Journal of Laboratory Medicine*.

[100] Buzanovskii V. A. (2017). Determination of proteins in blood. Part 1: determination of total protein and albumin. *Review Journal of Chemistry*.

[101] Vincent J.-L., Dubois M.-J., Navickis R., Wilkes M. (2003). Hypoalbuminemia in acute illness: is there aRationale for intervention?. *Annals of Surgery*.

[102] Chong J. J., Fragaszy E., Dukes O., Cason J., Kozlakidis Z. (2015). Serum albumin concentrations in a multi-ethnic cohort of patients with human immunodeficiency virus infection from south east London. *BioResearch Open Access*.

[103] Park J.-B., Kang D.-Y., Yang H.-M. (2013). Serum alkaline phosphatase is a predictor of mortality, myocardial infarction, or stent thrombosis after implantation of coronary drug-eluting stent. *European Heart Journal*.

[104] Kratz A., Ferraro M., Sluss P. M., Lewandrowski K. B. (2004). Normal reference laboratory values. *New England Journal of Medicine*.

[105] The Johns Hopkins Lupus Center (2017). Blood chemistry panel America. https://www.hopkinslupus.org/lupus-tests/screening-laboratory-tests/blood-chemistry-panel/.

[106] NIOS (2012). Kidney function test. *Biochemistry*.

[107] Strazzullo P., Leclercq C. (2014). Sodium. *Advances in Nutrition*.

[108] Shu Z., Tian Z., Chen J. (2018). HIV/AIDS-related hyponatremia: an old but still serious problem. *Renal Failure*.

[109] Arya S. S., Kumar P. K. (2012). Folate: sources , production and bioavailability. *Agro Food Industry Hi Tech*.

[110] Dieticians of Canada (2014). *Food Sources of Folate*.

[111] Bilski J., Mazur-Bialy A., Wojcik D. (2017). The role of intestinal alkaline phosphatase in inflammatory disorders of gastrointestinal tract. *Mediators of Inflammation*.

[112] D. Stead, Southern African HIV clinicians society guidelines, In: Health, South Africa, 2017

[113] WHO (2017). Guidelines for managing advanced HIV disease and rapid initiation of antiretroviral therapy. *HIV*.

