# Supplementary material for: Trends and Adaptive Optimal Set Points of CD4+ Count Clinical Covariates at Each Phase of the HIV Disease Progression
Source: AIDS Res Treat. 2020 Mar 1;2020:1379676. doi: 10.1155/2020/1379676 (PMC7068150; doi:10.1155/2020/1379676)
Supplement: Supplementary Materials — File S1: the raw data for the study. The data consist of 237 patients, who were followed up through four phases (2 to 5) post-HIV infection (excluding phase 1: HIV negative), where phase 2: acute infection is basically visits up to 3 months, monthly visits from 3 to 12 months (phase 3: early infection), quarterly visits thereafter (phase 4: established infection), and until ART initiation (phase 5). The observations are the repeated measurements of CD4+ count and 46 clinical covariates recorded during the last four visits of each phase. Figure S1: the contour plots of the complex optimal set points. The regions in peach correspond to desirable set points, whilst the blue regions represent the undesirable range of the clinical covariates. Figure S2: insignificant terms. Changes in these measurements does not have an influence on the CD4+ count. [file 1379676.f1.docx]

**File S1**: The raw data for the study. The data consist of 237 patients who were followed up through four phases (2 to 5) post HIV infection (excluding phase 1: HIV negative) where phase 2: acute infection is basically visits up to 3 months, monthly visits from 3 to 12 months (phase 3: early infection), quarterly visits thereafter (phase 4: established infection) until ART initiation (phase 5). The observations are the repeated measurements of CD4^+^ count and 46 clinical covariates recorded during the last four visits of each phase.


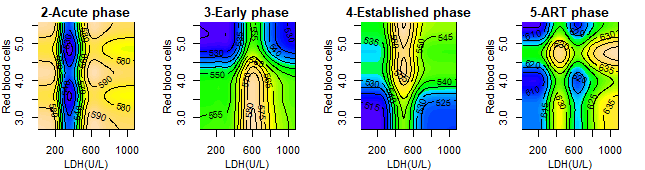


**Figure S1**: The contour plots of the complex optimal set-points. The regions in peach correspond to desirable set-points whilst the blue regions represent the undesirable range of the clinical covariates.


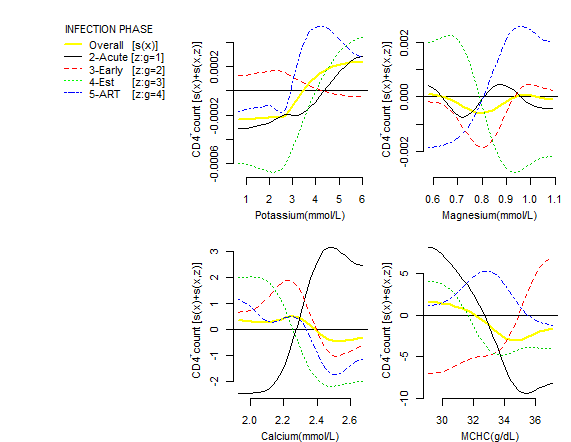


**Figure S2**: Insignificant terms. Changes in these measurements does not have an influence on the CD4^+^ count
